# *In vivo* functional characterisation of pheromone binding protein-1 in the silkmoth, *Bombyx mori*

**DOI:** 10.1038/s41598-018-31978-2

**Published:** 2018-09-10

**Authors:** Yusuke Shiota, Takeshi Sakurai, Takaaki Daimon, Hidefumi Mitsuno, Takeshi Fujii, Shigeru Matsuyama, Hideki Sezutsu, Yukio Ishikawa, Ryohei Kanzaki

**Affiliations:** 1 Research Center for Advanced Science and Technology, The University of Tokyo, 4-6-1 Komaba, Meguro-ku, Tokyo 153-8904 Japan; 2 Department of Agricultural Innovation for Sustainability, Faculty of Agriculture, Tokyo University of Agriculture, 1737 Funako, Atsugi, Kanagawa 243-0034 Japan; 3 Department of Applied Biosciences, Graduate School of Agriculture, Kyoto University, Kitashirakawa Oiwakecho, Sakyo-ku, Kyoto 606-8502 Japan; 4 Agricultural and Environmental Biology, Graduate School of Agricultural and Life Sciences, The University of Tokyo, 1-1-1 Yayoi, Bunkyo-ku, Tokyo 113-8567 Japan; 5 Graduate School of Life and Environmental Sciences, University of Tsukuba, 1-1-1 Tennodai, Tsukuba, Ibaraki 305-8572 Japan; 6 Transgenic Silkworm Research Unit, Institute of Agrobiological Sciences, National Agriculture and Food Research Organization, 1-2 Owashi, Tsukuba, Ibaraki 305-8634 Japan

## Abstract

Male moths detect sex pheromones emitted by conspecific females with high sensitivity and specificity by the olfactory sensilla on their antennae. Pheromone binding proteins (PBPs) are highly enriched in the sensillum lymph of pheromone sensitive olfactory sensilla and are supposed to contribute to the sensitivity and selectivity of pheromone detection in moths. However, the functional role of PBPs in moth sex pheromone detection *in vivo* remains obscure. In the silkmoth, *Bombyx mori*, female moths emit bombykol as a single attractive sex pheromone component along with a small amount of bombykal that negatively modulates the behavioural responses to bombykol. A pair of olfactory receptor neurons, specifically tuned to bombykol or bombykal, co-localise in the trichodeum sensilla, the sensillum lymph of which contains a single PBP, namely, BmPBP1. We analysed the roles of BmPBP1 using *BmPBP1*-knockout silkmoth lines generated by transcription activator-like effector nuclease-mediated gene targeting. Electroantennogram analysis revealed that the peak response amplitudes of *BmPBP1*-knockout male antennae to bombykol and bombykal were significantly reduced by a similar percentage when compared with those of the wild-type males. Our results indicate that BmPBP1 plays a crucial role in enhancing the sensitivity, but not the selectivity, of sex pheromone detection in silkmoths.

## Introduction

Male moths utilize sex pheromones emitted by conspecific females to identify and locate their mates^1,2^. To detect the minute amounts of sex pheromones that are diluted in the air, male moths have evolved a sophisticated olfactory system that can detect conspecific pheromones with extreme sensitivity and specificity. Molecular mechanisms underlying the detection and discrimination of sex pheromone components by male moths have been one of the major topics of research in the field of insect olfaction.

Sex pheromones emitted by female moths are detected by sex pheromone receptor proteins, which are expressed on the dendritic membrane of pheromone-specific olfactory receptor neurons (ORNs) in the sensilla trichodea located on the antennae of male moths^3,4^. These ORNs are bathed in an aqueous solution referred to as sensillum lymph. Because most pheromone molecules are highly hydrophobic, they are believed to be solubilised into the sensillum lymph and are transported to pheromone receptors after they bind with small soluble proteins (about 15 kDa) named pheromone binding proteins (PBPs) that are highly enriched in the sensillum lymph^4–6^. PBPs, which belong to the odorant binding protein (OBP) family of insects^7^, are expressed in accessory cells surrounding the ORNs and are secreted into the sensillum lymph of pheromone-sensitive olfactory sensilla^8^. In addition to the solubilisation of pheromones, PBPs have been proposed to participate in the discrimination of sex pheromone components based on the fact that each moth species possesses multiple PBPs that exhibit different binding affinities to different sex pheromone components, as has been demonstrated in *in vitro* binding assays using several moth species^9–11^.

The silkmoth *Bombyx mori* is one of the model insects used in sex pheromone communication research. Peripheral pheromone detection system of this species, including relationships among the sensillum types, ORNs, pheromone receptors, and PBPs, is well characterised^12^. Female silkmoths emit bombykol [(*E,Z*)-10,12-hexadecadien-1-ol] and bombykal [(*E,Z*)-10,12-hexadecadienal] from their sex pheromone gland at a typical ratio of 11:1^13^. Of these two compounds, only the major component, bombykol, is sufficient to induce pheromone source orientation behaviour in male moths^13,14^, whereas the minor component, bombykal, negatively modulates the initiation of this behaviour^13,15^. Male silkmoths detect these pheromones using long sensillum trichodea on their antennae^16^. This type of sensilla contain a pair of bombykol- and bombykal-sensitive ORNs that express sex pheromone receptors specific to bombykol (BmOR1) and bombykal (BmOR3), respectively^14–19^. In the silkmoth, one PBP gene (*BmPBP1*) and two PBP-like genes (*BmPBP2, 3*) have been reported^20,21^. However, only *BmPBP1* has been shown to be expressed in the sensillum lymph of pheromone sensitive sensilla and its associated accessory cells at mRNA and protein levels^21,22^, whereas BmPBP2 and BmPBP3 are expressed in accessory cells that are not associated with the pheromone-sensitive sensilla^21^. In addition, immunohistochemical analysis has shown that other OBPs that have been examined (GOBP1, GOBP2, and ABPX) are rarely or not expressed in the long sensilla trichodea of male antennae^22^. These findings suggest that BmPBP1 is involved in the detection of bombykol and bombykal whereas BmPBP2 and BmPBP3 play roles in the detection of compounds other than sex pheromones. However, the possible functions of BmPBP1 have yet to be conclusively established, particularly with respect to whether BmPBP1 plays a crucial role in the discrimination between bombykol and bombykal: BmPBP1 has been reported to be required for the response selectivity of BmOR1 expressed in HEK293T cells to bombykol^23^, whereas BmOR1 expressed in *Xenopus* oocytes and Sf21 cells is able to respond specifically to bombykol without BmPBP1. However, these results have yet to be verified by conducting *in vivo* functional analyses using *BmPBP1* gene knockout moths.

In the present study, we generated *BmPBP1*-knockout silkmoth lines by using transcription activator-like effector nuclease (TALEN)-mediated gene targeting in order to elucidate the *in vivo* functions of BmPBP1 in the sex pheromone detection of male silkmoths. By electroantennogram (EAG) recordings, we show that the response of *BmPBP1-*knockout male antennae to both bombykol and bombykal was significantly reduced compared with those in the wild-type moths. We also established that the sensitivity of the initiation of pheromone source orientation behaviour is reduced in *BmPBP1-*knockout male moths. On the basis of our results, we discuss whether BmPBP1 contributes to the sensitive and selective detection of bombykol and bombykal in male silkmoths.

## Results

### Establishment of *BmPBP1*-knockout silkmoths

**Figure 1:**
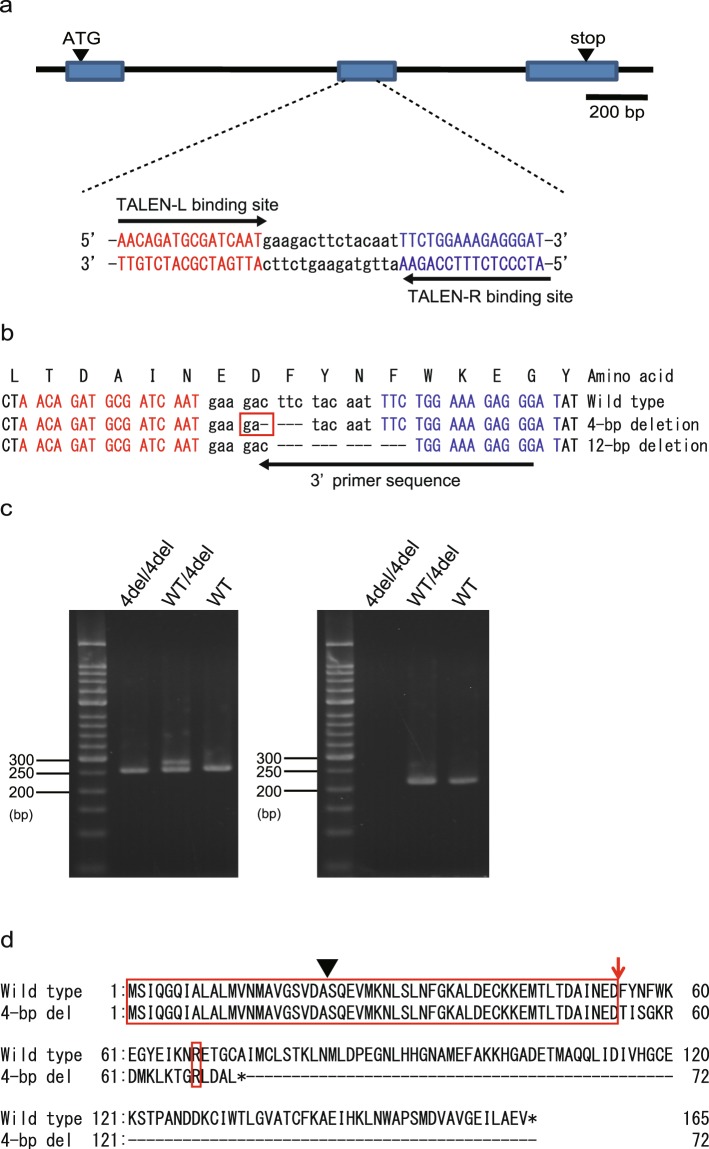
Generation of *BmPBP1*-knockout silkmoths. **(a)** Schematic representation of the genomic structure of *BmPBP1* (top) and target sequences of transcription activator-like effector nucleases (TALENs; bottom). Exons are indicated by blue boxes and the start/stop codon locations are shown. TALENs were constructed to target sequences in the second exon. The sequences of TALEN recognition sites are shown at the bottom of the genomic structure. **(b)** TALEN-induced mutant alleles generated in this study. The wild-type sequence is aligned with the deletion mutant sequences of *BmPBP1*. The deletions are indicated by a dashed line. The red box in the 4-bp deletion sequence indicates the position of a frame shift. Right and left TALEN recognition sequences are highlighted in red and blue characters, respectively. The black arrow under the sequences indicates the 3′ primer site used for genotyping by using genomic PCR. **(c)** A representative genomic PCR analysis of the 4-bp deletion allele is shown. The PCR products obtained using genomic DNA isolated from the wings of a mutation-homozygous individual (4del/4del), or a mutation-heterozygous individual (WT/4del), and a wild-type individual were separated by electrophoresis. The PCR primers corresponding to sequences flanking the deletion regions (left) or PCR primers with the 3′ primer designed to anneal to the deleted sequence (the black arrow in (b))(right) were used. **(d)** Deduced amino acid sequences of wild-type (top) and 4-bp deletion BmPBP1 moths (bottom). The black arrowhead on the sequences indicates a signal peptide cleavage site, and the red arrow indicates the position of a frame shift caused by the deletions. Red boxes indicate amino acids that are identical between the two sequences.

To characterise the functional role of PBPs in the detection of sex pheromones *in vivo*, we generated *BmPBP1*-knockout silkmoths using TALENs that were designed to target the second exon of the *BmPBP1* gene, which is located on the 19th chromosome (Fig. 1a). By genomic PCR screening of G_1_ individuals, followed by sequence analyses, we obtained two genetic lines with 4- and 12-bp deletions in the target region of the TALENs, respectively (Figs 1b and S1). From these two genetic lines, we selected a line that had the 4-base deletion. This deletion caused a frame shift at the 55th amino acid residue of the BmPBP1 protein and the introduction of a stop codon at the 73rd amino acid residue. Given that the first 22 amino acids of this protein constitute a signal peptide, this premature translational termination would result in a truncated protein of only 51 amino acids, compared with the 142 amino acids of the wild-type protein^20^. The mutant BmPBP1 protein contained only three out of the nine residues that form the bombykol binding pocket and only one out of the six cysteine residues required for proper folding^24,25^, resulting in the synthesis of a loss-of-function BmPBP1 protein (Fig. 1c).

To confirm the 4-bp deletion at the transcript level, we amplified full-length coding sequences of *BmPBP1* from the antennal cDNA of homozygous mutant (*BmPBP1−/BmPBP1−*) male moths using RT-PCR. After cloning the PCR products into a sequence plasmid vector, we sequenced 11 clones and confirmed that the DNA insert in all the clones had the same deletion as that in the genomic sequence, further supporting our observation that this allele encodes a substantially truncated BmPBP1 protein. We noted that both *BmPBP1−/BmPBP1−* female and male moths were fertile, and that their offspring grew normally to adulthood.

### EAG analysis of *BmPBP1-*knockout male antennae

**Figure 2:**
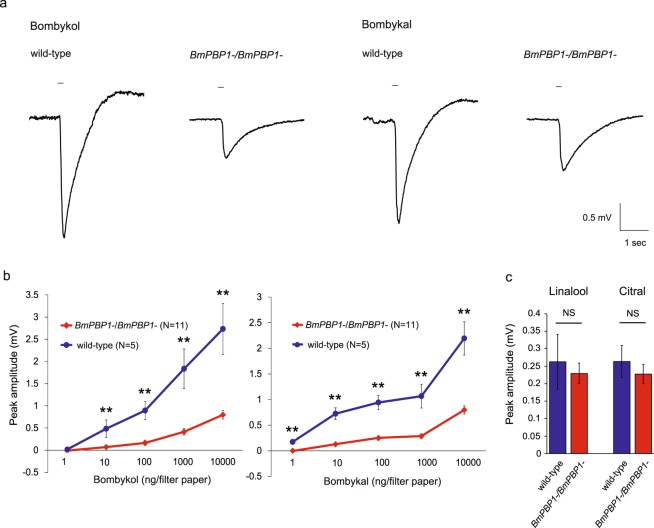
Electroantennogram (EAG) analyses of the response of BmPBP1 mutants to sex pheromone components. **(a)** Representative EAG of the antennae from *BmPBP1−/BmPBP1−* and wild-type male moths in response to 1000 ng bombykol (left) and bombykal (right). The stimulus was applied for 200 ms, as indicated by the solid line on the trace. **(b)** Dose-dependent increase in bombykol- (left) or bombykal- (right) induced peak EAG amplitudes in *BmPBP1−/BmPBP1−* (red; n = 11) and wild-type (blue; n = 5) male moth antennae. Error bars represent ± SEM. The asterisks indicate significant differences between the groups (**p < 0.01), as determined using Student’s *t*-test for comparing pairs of data. **(c)** Comparison of linalool and citral-induced peak EAG amplitudes of *BmPBP1−/BmPBP1−* (red; n = 5 for linalool, n = 6 for citral) and wild-type (blue; n = 5 for linalool, n = 7 for citral) male moths. Error bars represent ± SEM. No significant difference was detected between the two groups (Student’s *t*-test; *p* = 0.700 for linalool, *p* = 0.529 for citral).

To determine the effects of the loss of function of BmPBP1 on the olfactory response in male moths, we recorded the EAG responses of male antennae to sex pheromone components. The peak EAG amplitudes of the antennae of *BmPBP1-*knockout males to bombykol and bombykal were significantly reduced compared with those of the antennae of wild-type males (Fig. 2a,b). Notably, the degree of reduction in EAG amplitude was similar between the responses to bombykol and bombykal (Table 1), suggesting that BmPBP1 contributes to the detection of these two components to a similar extent, and thus is not likely to have selectivity for either of the two compounds. Although the peak amplitudes were significantly lower than those of the wild-type males, antennae from the *BmPBP1*-knockout males showed clear dose-dependent responses to bombykol and bombykal (Fig. 2a,b), suggesting that male moths can detect bombykol and bombykal, albeit with low sensitivity, in the absence of functional BmPBP1.

**Table 1:** Percentage reduction in the peak electroantennogram (EAG) amplitudes in the *BmPBP1*-knockout male moths.

Stimulation	10 ng	100 ng	1000 ng	10000 ng
Bombykal	82.2%	73.3%	73.1%	63.7%
Bombykol	85.6%	81.9%	77.4%	70.8%

To exclude the possibility that the loss of BmPBP1 affected the olfactory response of antennae as a whole, we analysed the EAG response of male antennae to general odorants, linalool and citral^26^. The peak EAG amplitudes for the response to both linalool and citral were not significantly different between the antennae of the *BmPBP1*-knockout and wild-type males (Figs 2c and S2). Although linalool reportedly inhibits most of bombykol receptor neurons and activates some of them^27^, our results that *BmPBP1*-knockout did not affect EAG responses to general odorants indicate the lowered response of *BmPBP1*-knockout male moths to pheromones was not due to a general effect on olfactory detection.

### Behavioural analysis of *BmPBP1*-knockout male moths

**Figure 3:**
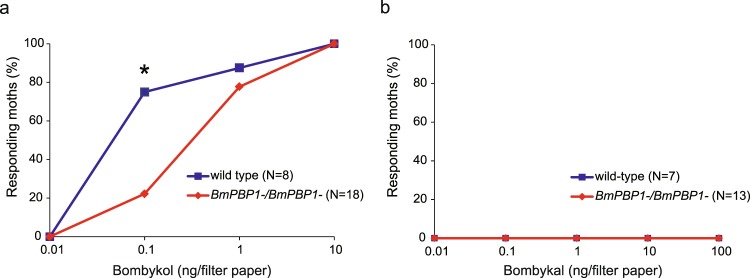
Behavioural response of *BmPBP1*-knockout males to pheromones. The behavioural response percentages of *BmPBP1−/BmPBP1−* (red) and wild-type (blue) male moths to different doses of bombykol **(a)** or bombykal **(b)** are plotted. The asterisk indicates significant differences between the groups (*p < 0.05), as determined using Fisher’s exact probability test for comparing pairs of data.

Finally, using a closed-box assay^28,29^, we investigated whether the reduced response of antennae to bombykol caused by *BmPBP1* mutation affected the behavioural responsiveness to bombykol. In this assay, we used wing flapping behaviour as a criterion for initiation of the pheromone source orientation behaviour. Dose-response analysis revealed that the percentage of *BmPBP1*-knockout males that initiated the pheromone source orientation behaviour was lower than that of the wild-type males (Fig. 3a). As in wild-type males, *BmPBP1*-knockout males did not show any behavioural responses to bombykal even at the highest dose tested (100 ng; Fig. 3b). These results indicate that the behavioural responsiveness to bombykol was reduced in *BmPBP1*-KO males.

## Discussion

In this study, we demonstrated that the loss of functional BmPBP1 led to the lowering of EAG responses to both bombykol and bombykal to a similar extent. These observations indicate two important aspects of BmPBP1 function: (1) BmPBP1 is necessary for the sensitive detection of bombykol and bombykal and (2) BmPBP1 is not involved in the discrimination between bombykol and bombykal. Our results are consistent with those of a recent study by Ye *et al*., wherein the authors observed significant reductions in the EAG responses of the antennae of *PBP1*-knockout *Helicoverpa armigera* males to three sex pheromone components in this species^30^.

Previous studies suggested two different molecular mechanisms underlying the specific response of male silkmoths to bombykol. On the basis of an *in vivo* opened sensillum tip analysis, Pophof reported that BmPBP1 mediates the response of BmOR1 to bombykol but not to bombykal, and thus proposed that response selectivity to pheromones is intermediated by PBPs specialized in the recognition of particular ligands^31^. Using HEK293T cell expression system, Große-Wilde *et al*. showed that BmOR1 was able to respond to both bombykol and bombykal when these compounds were solubilised by dimethyl sulfoxide (DMSO), but responded only to bombykol when BmPBP1 was used as a solubiliser instead of DMSO^23^, suggesting that BmPBP1 selectively solubilised bombykol and that the interplay between bombykol and BmPBP1 is important for the selective response of BmOR1 to bombykol. Accordingly, these authors proposed that other PBPs that bind to bombykal and mediate the bombykal response should be present in the sensilla trichodea of male moths. In contrast, it has been demonstrated that *Xenopus* oocytes or Sf21 cells, co-expressing BmOR1 and the co-receptor BmOrco, responded specifically to bombykol that was dissolved in DMSO, suggesting that specific interaction between bombykol and BmOR1 defines the specificity of the response^18,32^. Our results, demonstrating that BmPBP1 mediates the response to both bombykol and bombykal *in vivo*, are consistent with the latter mechanism because a BmOR1-expressing bombykol-sensitive ORN and a BmOR3-expressing bombykal-sensitive ORN co-localise in the same sensilla in the silkmoth, and are thus bathed in the same sensillum lymph containing BmPBP1^22^. Further, our results are consistent with the docking simulation and *in vitro* binding assay reported by Gräter *et al*., which showed that BmPBP1 bound to both bombykol and bombykal with nearly the same affinity^33^.

Although the responsiveness of the antennae of *BmPBP1*-knockout males to sex pheromones was considerably lower than that of the antennae of the wild-type males, *BmPBP1*-knockout male antennae still showed dose-dependent EAG responses to bombykol and bombykal. Therefore, even in the absence of a functional BmPBP1, subsets of pheromone molecules can reach the dendritic membranes of ORNs and activate pheromone receptor proteins expressed on the membranes. The residual EAG response of the antennae of *BmPBP1*-knockout male moths may be explained by the non-specific binding and solubilisation of the pheromone molecules with some water soluble proteins in the sensillum lymph. In this regard, we cannot exclude the possibility that other OBPs are co-expressed with BmPBP1 in the sensillum trichodea. Although we cannot completely exclude the possibility that the mutant BmPBP1 still has some binding capacity to the pheromones, it’s highly unlikely because mutant BmPBP1 protein doesn’t contain amino acid residues required for proper folding and formation of the binding pocket for bombykol (see Results). Alternatively, there may be another pathway from the olfactory pore to the pheromone receptors, and in this regard, it has been reported that at least some of the olfactory tubules in the sensillum trichodea come into contact with the dendritic membrane of ORNs in the giant silkmoth *Antheraea polyphemus*, thereby raising the possibility of a direct pathway from the olfactory pores to the dendritic membranes^34,35^. Further studies are accordingly required to clarify how pheromone molecules reach the pheromone receptors in the absence of solubilisation by BmPBP1.

We also demonstrated the effects of *BmPBP1*-knockout at the behavioural level, showing that the percentage of male moths that initiate pheromone source orientation behaviour was significantly reduced in *BmPBP1*-knockout males. To gain a more precise understanding of the functional role of BmPBP1 in the sex pheromone communication system, it would be informative to examine not only the sensitivity of behavioural response initiation but also the efficiency of male orientation to female moths and successful copulation.

In summary, by using *BmPBP1*-knockout moths, we showed that BmPBP1 contributes to the sensitivity of pheromone detection, but does not play a significant role in the discrimination of bombykol and bombykal. Apart from the solubilisation and transportation of pheromones, PBPs have also been proposed to play additional roles^36^, including the protection of pheromone molecule from enzymatic degradation^37^ and rapid inactivation of pheromones^38^. Detailed physiological analyses of *BmPBP1*-knockout moths will shed further light on the underlying mechanisms and modes of action of BmPBP1 in pheromone detection *in vivo*.

## Methods

### Animals and chemicals

The *w1-pnd* strain of *Bombyx mori*, which is non-diapausing and characterised by non-pigmented eggs and eyes, was used for the generation of *BmPBP1*-knockout moths. Larvae were reared on an artificial diet (Nihon Nosan Kogyo, Yokohama, Japan) at 25 °C under a 16:8 h (light/dark) photoperiod. The purity (>99.5%) of synthetic bombykol and bombykal were verified by gas chromatography under previously described conditions^39,40^.

### Construction of TALEN vectors and synthesis of RNA for injection

TALEN expression vectors were constructed as described previously^41,42^. The RNA used for injection was prepared as described previously^28^ using a Qiagen Hispeed plasmid midi kit (Qiagen, Hilden, Germany) and an mMESSAGE mMACHINE T7 Ultra Transcription kit (Ambion, Austin, USA). Extracted RNA was purified by LiCl precipitation, followed by four washes with 70% ethanol. The RNA of left and right TALENs was dissolved at a concentration of 0.2 μg/μL in 0.5 mM phosphate buffer (pH 7.0) containing 5 mM KCl, and this RNA solution was injected into preblastoderm-stage embryos, as described previously^43^.

### Screening of mutagenised moths

G_1_ eggs were obtained by the sibling mating of G_0_ adults. Genomic DNA of G_1_ eggs from different broods was extracted separately using a DNeasy Blood & Tissue Kit (Qiagen, Hilden, Germany). The region surrounding the target site was PCR amplified using genomic DNA as the template and specific primers (sense: 5′-CGACCTTCGCAAGGTATGAT-3′ and antisense: 5′-AGGCACATTATAGCGCATCC-3′). The PCR products were sequenced directly using an ABI3700 DNA analyser (Applied Biosystems, Foster City, CA, USA). G_1_ broods that showed overlapping sequencing patterns with the target sequence were reared to the adult stage. After the G_1_ moths had been crossed with wild-type adults, their genomic DNA was extracted, PCR amplified, and sequenced, as described above, to identify the mutagenised individuals. *BmPBP1−/BmPBP1−* males were obtained by crossing *BmPBP1−/BmPBP1*+ females with *BmPBP1−*/*BmPBP1*+ males.

### Reverse-transcription-PCR

Total RNA was extracted from male moth antennae using TRIzol reagent (Invitrogen, Carlsbad, CA, USA) as described previously^29^. The extracted RNA was reverse transcribed using an oligo(dT) adaptor primer (Takara-Bio, Otsu, Japan) and AMV reverse transcriptase (Takara-Bio, Otsu, Japan) at 42 °C for 30 min. The cDNA of *BmPBP1* was amplified using Ex *Taq* DNA polymerase (Takara-Bio, Otsu, Japan) and a primer pair for *BmPBP1* (5′-ATGTCTATCCAAGGACAGATCGC-3′ and 5′-TCAAACTTCAGCTAAAATTTCTCCC-3′), with thermal cycling at 95 °C for 5 min, followed by 30 cycles at 95 °C for 30 s, 53 °C for 30 s, and 72 °C for 30 s, and a final extension at 72 °C for 5 min. Equal amounts of PCR products were separated by electrophoresis on 2.0% agarose gels. The cDNA was cloned into a pGEM-T Easy vector (Promega, Madison, USA), and the PCR products were sequenced using an ABI3700 DNA analyser (Applied Biosystems, Foster City, CA, USA).

### Electroantennogram (EAG) recordings

The antennae of male moths were excised at the base, and a few segments at the tip were cut off. The antennae were then mounted on the EAG probe using electrode gel (SPECTRA 360; Parker Laboratories, Fairfield, NJ, USA). A glass cartridge (inner diameter, 5 mm) was prepared for stimulation by inserting a piece of filter paper (1.5 × 1.5 cm), and 5 μL of pheromone solution in *n*-hexane or neat *n*-hexane (control) was administered. For general odorant stimulation, 5 μL of 10% (v/v) linalool or citral (Wako, Osaka, Japan) in paraffin oil (Sigma-Aldrich, St. Louis, USA) was used as the stimulant. A charcoal-purified airstream (1 L/min) was passed through a glass pipette and directed onto the antenna. The EAG responses were amplified using a custom-made amplifier (Minegishi and Kanzaki, unpublished), low-pass filtered at 300 Hz, and digitised at 1 kHz (USB-6210; National Instruments, Austin, TX, USA). The data were analysed using a custom-written programme (MATLAB; Mathworks, Natick, MA, USA). Following EAG measurements, the genotype of all males was confirmed by PCR as described in the section ‘Screening of mutagenised moths’. The percentage reduction in peak EAG amplitude (Table 1) was calculated by dividing the peak EAG amplitude of *BmPBP1−/BmPBP1−* male antennae by that of wild-type males.

### Behavioural experiments

The behavioural responses to bombykol were examined as described previously^29^, with the exception that the male silkmoths were used within 1 to 5 days after eclosion. The moths were exposed to increasing doses of bombykol or bombykal (0.01, 0.1, 1, and 10 ng) at 1-min intervals. Wing flapping within 10 s of the stimulation that lasted for more than 10 s was counted as a response. Subsequent to these behavioural experiments, the genotype of all the males was determined by PCR.

### Statistical analysis

To assess the statistical significance of differences in the EAG and behavioural response data for wild-type and *BmPBP1*-knockout moths, we used Student’s *t*-test and Fisher’s exact probability test for comparing pairs of data, respectively, by using Microsoft Excel 2010 and a commercial macroprogramme (Statcel version 3; Seiun-sya, Japan). The error bars shown in figures represent SEMs.

## Electronic supplementary material

Supplementary Figure
