# Supplementary material for: In vivo functional characterisation of pheromone binding protein-1 in the silkmoth, Bombyx mori
Source: Sci Rep. 2018 Sep 10;8:13529. doi: 10.1038/s41598-018-31978-2 (PMC6131395; doi:10.1038/s41598-018-31978-2)
Supplement: Supplementary file 1 — Supplementary Figure [file 41598_2018_31978_MOESM1_ESM.pdf]

In vivo functional characterisation of pheromone binding protein-1 in the silkmoth, *Bombyx mori*

Yusuke Shiota[1], Takeshi Sakurai[1,2]*, Takaaki Daimon[3], Hidefumi Mitsuno[1], Takeshi Fujii[4], Shigeru Matsuyama[5], Hideki Sezutsu[6], Yukio Ishikawa[4] & Ryohei Kanzaki[1]

[1]Research Center for Advanced Science and Technology, The University of Tokyo, 4-6-1 Komaba, Meguro-ku, Tokyo 153-8904, Japan

[2]Department of Agricultural Innovation for Sustainability, Faculty of Agriculture, Tokyo University of Agriculture, 1737 Funato, Atsugi, Kanagawa 243-0034, Japan

[3]Department of Applied Biosciences, Graduate School of Agriculture, Kyoto University, Kitashirakawa Oiwakecho, Sakyo-ku, Kyoto 606-8502, Japan

[4]Agricultural and Environmental Biology, Graduate School of Agricultural and Life Sciences, The University of Tokyo, 1-1-1 Yayoi, Bunkyo-ku, Tokyo 113-8567, Japan

[5]Graduate School of Life and Environmental Sciences, University of Tsukuba, 1-1-1 Tennodai, Tsukuba, Ibaraki 305-8572, Japan

[6]Transgenic Silkworm Research Unit, Institute of Agrobiological Sciences, National Agriculture and Food Research Organization, 1-2 Owashi, Tsukuba, Ibaraki 305-8634, Japan

*Corresponding author

Takeshi Sakurai, Ph.D.

Department of Agricultural Innovation for Sustainability, Faculty of Agriculture, Tokyo University of Agriculture, 1737 Funako, Atsugi, Kanagawa 243-0034, Japan

TEL: +81-46-270-6124, Fax: +81-46-270-6124,

E-mail: ts206448@nodai.ac.jp

Fig. S1

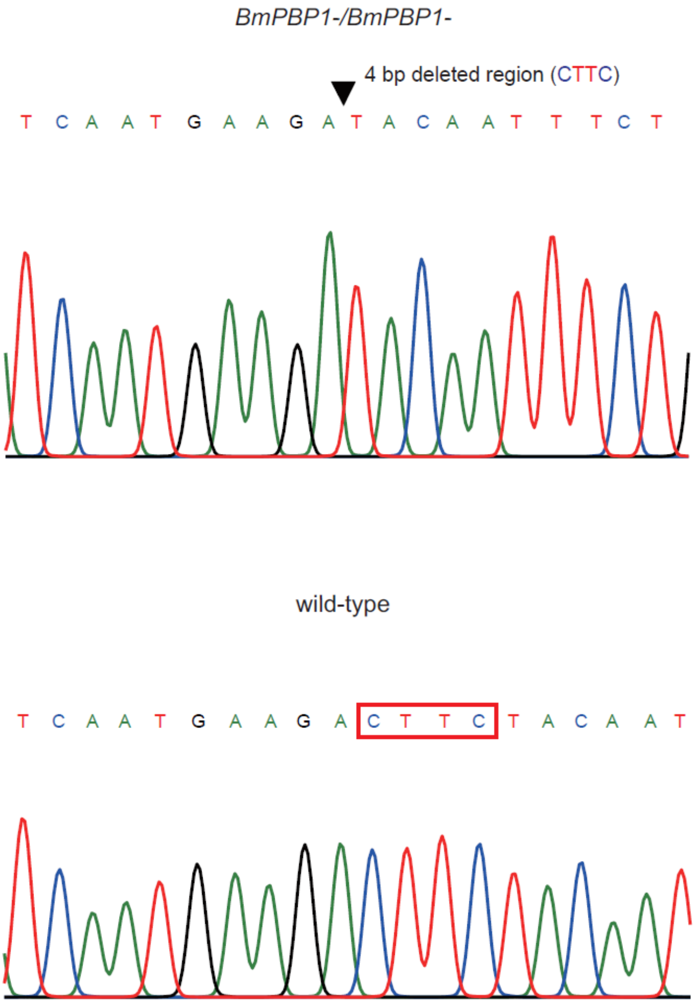

Fig. S1
Representative DNA sequencing results for *BmPBP1-/BmPBP1-* and wild-type male around the 4-bp deleted region in the *BmPBP1* gene of *Bombyx mori*.

Fig. S2

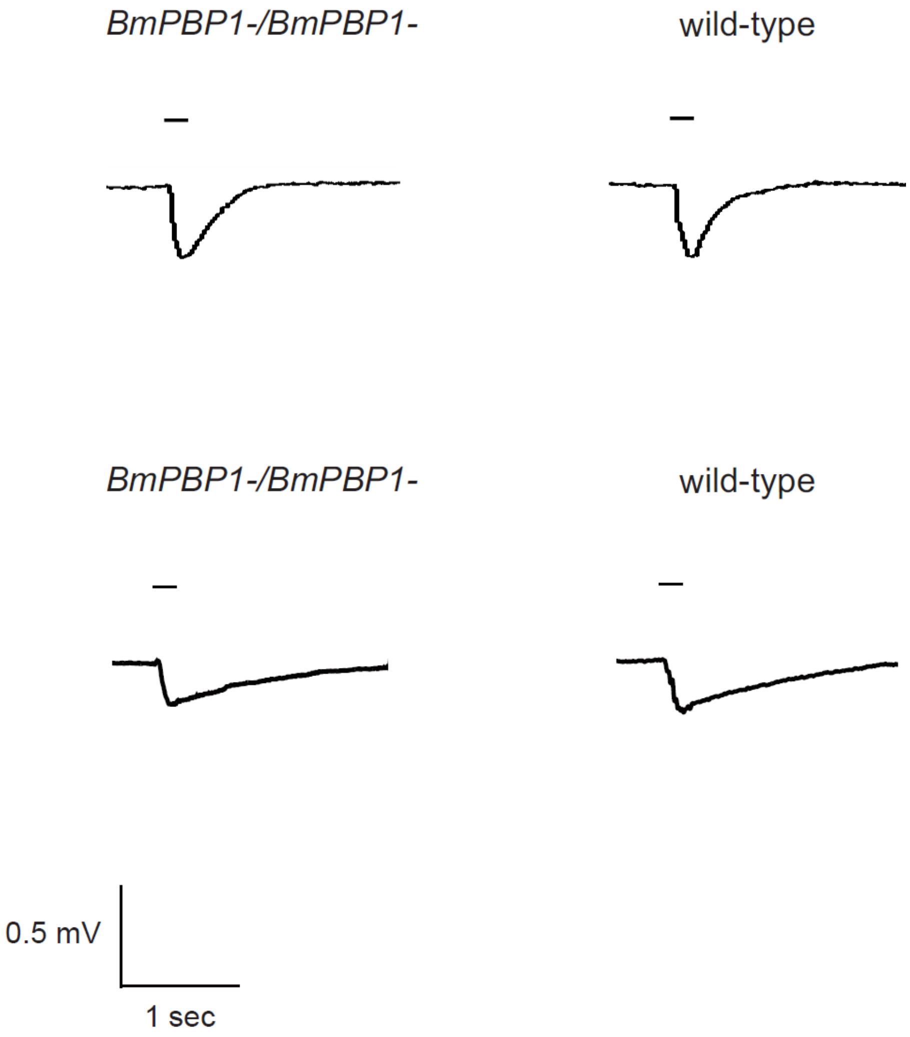

Fig. S2
Representative electroantennogram (EAG) responses of *BmPBP1-/BmPBP1-* and wild-type male antennae to 10% linalool (top) and citral (bottom). The stimulus was applied for 200 ms, as indicated by the solid line on the trace.
